# Further development in measuring communicative participation: identifying items to extend the applicability of the communicative participation item bank

**DOI:** 10.1186/s41687-023-00586-8

**Published:** 2023-05-26

**Authors:** Nicole ter Wal, Lizet van Ewijk, Johanna M.A. Visser-Meily, Anna Volkmer, Ellen Gerrits, Caroline B. Terwee

**Affiliations:** 1 Research Centre Healthy and Sustainable Living, HU University of Applied Sciences Utrecht, P.O. box 12011, Utrecht, 3501 AA The Netherlands; 2 Department of Languages, Literature and Communication, Utrecht Institute of Linguistics OTS, Utrecht University, Utrecht, The Netherlands; 3 Department of Rehabilitation, Physical Therapy Science & Sports, UMC Utrecht Brain Centre, University Medical Centre Utrecht, Utrecht, The Netherlands; 4 Centre of Excellence for Rehabilitation Medicine, UMC Utrecht Brain Centre, De Hoogstraat Rehabilitation, University Medical Centre Utrecht and Utrecht University, Utrecht, The Netherlands; 5 Department of Language and Cognition, University College London, London, UK; 6 Department of Epidemiology and Data Science, Amsterdam Public Health research institute, Amsterdam UMC, Vrije Universiteit Amsterdam, Amsterdam, The Netherlands

**Keywords:** Communicative participation, Patient reported outcome measure, Language problems, Voice problems, Hearing problems, Speech problems

## Abstract

**Background:**

The ability to communicate is a prerequisite for participation in today’s society. To measure participation in adults with communication disorders, the Communicative Participation Item Bank (CPIB) was developed in 2006. Since then, several new PROMs have been developed to measure communication and the impact of communication disorders on participation. Moreover, the CPIB items do not all appear to be relevant to certain populations with communication problems and context of communicative participation is changing rapidly, given the increased use of digital communication forms. The purpose of this study was to identify new PROMs developed since 2006 that aim to measure (aspects of) communication, in order to select items that are suitable for expanding the Communicative Participation Item Bank to make the item bank more widely applicable (e.g., to the hearing-impaired population) and tailored to the current societal context.

**Methods:**

Medline and Embase were used to search for PROMs that aim to measure (aspects of) communication. Each new PROM as well as the CPIB, was evaluated to determine to what extent it contains items that measure communicative participation and to what extent these items capture all communicative participation domains by linking each item to the ICF Activities and Participation domains.

**Results:**

This study identified 31 new PROMs, containing 391 items that were labelled as measuring communicative participation. The majority of the 391 items measure aspects of ICF Activities and Participation domain ‘communication’, followed by the domain ‘interpersonal interactions and relationships’. The other ICF Activity and Participation domains were less often addressed. Analysis of the CPIB showed that items do not cover all domains of participation as defined in the ICF, such as the ‘major life areas’ domain.

**Conclusions:**

We found a potential pool of 391 items measuring communicative participation that could be considered for extending the CPIB. We found items in domains that are already present in the CPIB, but also items that relate to new domains, such as an item on talking with customers or clients for the ‘major life areas’ domain. Inclusion of new items in other domains would benefit the comprehensiveness of the item bank.

**Supplementary Information:**

The online version contains supplementary material available at 10.1186/s41687-023-00586-8.

## Introduction

Interpersonal communication is essential for successful participation in almost all aspects of life, ranging from family life to work, leisure, and education [1, 2]. It enables people to exchange information, express their needs and wishes and interact with others [3]. The importance of communication is perhaps best captured by Ruben who, in his paper ‘*Redefining the Survival of the Fittest*’ suggests that “the fitness of the person of the 21st century will be defined, for the most part, in terms of their ability to communicate effectively”, as communication plays a major role in the way people make their livelihoods [4].

Speech and language therapists (SLTs) provide treatment, support and care for people who have difficulties with communication, with increased participation being one of the fundamental outcomes of therapy [5–10]. Patient perspective on this outcome can be captured using Patient Reported Outcome Measures (PROMs) [11, 12] which can, in turn, provide unique information to further guide patient care [13]. In the Netherlands, however, existing instruments that aim to measure ‘participation’ are not specific or sensitive enough to capture (changes in) participation for people with different communication disabilities, since they do not explicitly address participation problems that are related to communication [2].

To focus on the specific communication difficulties associated with participation, Eadie et al. [2] introduced the construct of communicative participation. They defined this construct as “participation in life situations in which knowledge, information, ideas or feelings are exchanged” and added that “it may take the form of speaking, listening, reading, writing, or nonverbal means of communication [2]. ‘Communication’ and ‘participation’ are complex constructs that both have a range of definitions proposed in the literature [3, 14]. Regardless of the specific definition used, ‘communicative participation’ is proposed as the construct that emerges from the area where communication and participation overlap [15]. Communicative participation is a construct intended to exclude basic tasks related to body functions and structures (e.g., intelligibility of speech sounds, or hearing speech sounds), as well as activities where there is no exchange or opportunity for a response involved and those that do not usually occur in the context of a life situation (e.g., picture naming) [2].

In 2006, Eadie and colleagues reviewed existing self-report instruments in speech and language outcome research targeting communicative functioning, in search of an instrument that would measure communicative participation, or at least individual items that captured this construct. Six instruments were evaluated for the extent to which they measured the construct communicative participation. First, Eadie et al. [2] assessed all items on their fulfillment of the criterion, reflecting the construct of communicative participation “including a communicative exchange between at least two communicative partners (i.e., a message with the opportunity for a response) in the context of a life situation” [2]. Subsequently, the extent to which the instruments measured communicative participation in all its breadth was assessed, by linking each instrument’s individual item to one of their proposed communicative participation domains [2, 15]. These domains are based on the Activities and Participation domains of the International Classification of Functioning, Disability and Health (ICF) [16], although Eadie et al. used different names personal care, household management, work/education, leisure/recreation, relationships, and community [2]. An additional domain, general communication, was used to describe items that could cross multiple domains [2]. Eadie and colleagues concluded that none of the existing instruments fully covered the construct, and only 34 out of 132 items they reviewed addressed communicative participation. They subsequently developed a PROM to measure communicative participation in community-dwelling adults, based on items found in their literature review, interviews of participants with communication disorders and items created by a panel of experts [1, 17–19]: the Communicative Participation Item Bank (CPIB; [20]).

An item bank is a large set of questions, or items, which are all related to the same construct [21]. The items are ranked based on their ‘difficulty’ (referring to the level of the construct they address) and their discriminative ability using Item Response Theory (IRT) modeling. Therefore, item banks are suitable for generating various short forms or can be administered using computer adaptive testing (CAT), which means the test adapts in real time to the patient’s responses to items (i.e. the following item is selected based on the answer to previously answered items). An additional advantage of IRT-calibrated item banks is that they allow items to be added or removed without jeopardizing comparability with previous versions of the questionnaire [22]. Therefore, they are seen as the future of outcome measurement and preferred over the development of new static questionnaires [23–25].

Since 2006, several new PROMs have been developed to measure communication and the impact of communication disorders on participation. The context of communicative participation is changing rapidly, given the increased use of digital communication forms such as smart phones, social media, and work-related platforms such as Zoom or Teams [26, 27]. Therefore, it could be relevant to consider adding new content to the CPIB. Moreover, the CPIB items do not all appear to be relevant to certain populations, such as the population with hearing loss [15, 28]. This raises the question whether the current CPIB could and should be updated, tailored to the current societal context.

The CPIB is already used in several countries and is being translated into other languages [15]. Recently, the short form of the CPIB was translated into Dutch and validated in adults with speech problems due to a neurological aetiology or head and neck cancer [29]. The aim of this study was to identify new PROMs developed since 2006 aimed at measuring (aspects of) communication, in order to select items that are suitable for expanding the CPIB to make the item bank more widely applicable.

## Methods

### Phase 1: identification of relevant PROMs

#### Search strategy

In accordance with the COnsensus-based Standards for the selection of health Measurement INstruments (COSMIN) guideline for systematic reviews [30], a systematic search was performed in MEDLINE (via PubMed) and EMBASE, in order to identify PROMs designed to measure communication in an adult population and that had been validated to some extent. It was expected that by including PROMs designed to measure (aspects of) communication), all items would be found that meet the criterion for communicative participation (i.e. the overlap between communication and participation). We chose to target our search to literature from 2006 to December 7 2021^1^, since Eadie et al. [2] searched for instruments aimed at communication developed up to 2006 in their review.

The search strategy was created in consultation with a clinical librarian and comprised terms subsumed under four key elements of a review: (1) construct, (2) population, (3) type of instrument, and (4) measurement properties [30]. Several key words were used for a search string; *communication, PROM, adults with communication problems* and *measurement properties*, using a methodological search filter for finding measurement instruments [31]. The complete search strategy can be found in Appendix 1,^2^. The search targeted literature that described PROMs, i.e. all PROMs included in the review had to have a published study associated with it.

In addition to the literature search, the following online databases were searched to find additional relevant PROMs: Rehabilitation Measures Database [32], the PROQOLID database [33], Measuring instruments in healthcare database [34] and the COSMIN database of systematic reviews [35].

#### Screening abstracts and full text articles

Abstracts were included if: (1) They described an instrument aimed to measure communication, including instruments that aim to measure the impact of communication difficulties on daily life, or quality of life. (2) The instrument described was a PROM (i.e. self-reported) that had to be completed by an adult with communication problems. (3) There was information available on the measurement properties (i.e. on the development, validity, reliability, or other measurement properties). This inclusion criterion was used to exclude PROMs without psychometric evidence. (4) The article was written in English or Dutch.

Abstracts were excluded if: (1) The article was a review (except reviews of communication instruments). (2) The PROM described was developed before 2006. (3) The PROM described was aimed at measuring speech, voice, and hearing as a body function. These functions are required for communication, but do not describe communicative participation [2].

Full-text articles were then obtained and selected according to the same inclusion and exclusion criteria as listed for the abstracts. Two authors (NW: PhD-student and SLT and AV: Senior researcher and SLT) screened the abstracts, articles, and websites. When in doubt, a third author was consulted (LE: Senior researcher and SLT).

The names of the described PROMs were then extracted from the included articles and after deduplication entered in Microsoft Excel 365 [36]. Subsequently, a search was performed to find the PROM itself. For selected PROMs that were not freely available or could not be found, a request was sent to the developers.

To identify items that fit the construct of communicative participation, all selected PROMs were first independently screened by two authors (NW and LE) as containing at least some items measuring communication. For this process, the description of communication according to Eadie et al [2] was used: “knowledge, information, ideas or feelings are exchanged” and “may take the form of speaking, listening, reading, writing, or nonverbal means of communication”. When in doubt, a third author was consulted (AV). Secondly, in order to select items that could be suitable for expanding the CPIB, each item of the included PROM was analyzed (Phase 2).

### Phase 2: Identification of relevant items measuring the construct of communicative participation

For the selected PROMs, the items were assessed on the criterion for measuring communicative participation as used by Eadie et al. [2]. Items were considered relevant for measuring communicative participation when addressing “a communicative exchange between at least two communicative partners (i.e., a message with the opportunity for a response) in the context of a life situation” [2]. “A message with the opportunity for a response” implies that there is a natural communication partner who has the opportunity to respond immediately. This message can be a verbal message through spoken or written language, or a nonverbal message [2]. This inclusion criterion intends to exclude basic tasks related to body functions and structures, such as ‘hearing sounds’, as well as activities where there is no exchange or opportunity for a response involved, such as ‘listening to the radio’ or ‘watching television’ [2]. Two authors independently assessed all PROM items (NW and LE). Differences were discussed until consensus was reached.

The items labelled as ‘measuring communicative participation’ were then classified into different domains. Since communicative participation situations occurs in those ICF Activities and Participation domains in which knowledge, information, ideas, or feelings are exchanged [2, 15], linking the items to the relevant ICF Activities and Participation domains may identify gaps in the existing item bank and newly identified items. As described in the introduction, Eadie et al. [2] considered six of the nine ICF Activities and Participation domains relevant for the construct of communicative participation (see Table 1) [2].

**Table 1:** ICF Activities and Participation domains and domains used by Eadie et al. [2]

ICF Activities and Participation domains [16]	Domains used by Eadie et al. [2]
1) **Learning and applying knowledge** *(“learning, applying the knowledge that is learned, thinking, solving problems, and making decisions”)*	
2) **General tasks and demands** *(“general aspects of carrying out single or multiple tasks, organizing routines and handling stress”)*	
3) **Communication** *(“general and specific features of communicating by language, signs and symbols, including receiving and producing messages, carrying on conversations, and using communication devices and techniques”)*	**General communication** *(“general communication items that could cross multiple domains”)*
4) **Mobility** *(“moving by changing body position or location or by transferring from one place to another, by carrying, moving or manipulating objects, by walking, running or climbing, and by using various forms of transportation”)*	
5) **Self-care** *(“caring for oneself, washing and drying oneself, caring for one’s body and body parts, dressing, eating and drinking, and looking after one’s health”)*	**Personal care** *(“communicating in situations related to self-care”)*
6) **Domestic life** *(“carrying out domestic and everyday actions and tasks. Areas of domestic life include, caring for one’s belongings and space, acquiring food, clothing and other necessities, household cleaning and repairing, caring for personal and other household objects, and assisting others”)*	**Household management** *(“communicating in situations related to performing the routine duties of managing a household and those living together in it”)*
7) **Interpersonal interactions and relationships** *(“carrying out the actions and tasks required for basic and complex interactions with people (strangers, friends, relatives, family members and lovers) in a contextually and socially appropriate manner”)*	**Relationships** *(“communicating in situations that connect or bond participants, including family, friends, and romantic relationships”)*
8) **Major life areas** *(“carrying out the tasks and actions required to engage in education, work and employment and to conduct economic transactions”)*	**Work/education** *(“communicating in situations related to paid or unpaid (volunteer) employment or school work”)*
9) **Community, social and civic life** *(“actions and tasks required to engage in organized social life outside the family, in community, social and civic areas of life”)*	**Community** *(“communicating in situations related to community integration”)* **Leisure/recreation** *(“communicating in situations related to discretionary activities not related to work or other duties; may be either quiet or active activities”)*

We argue that communicative participation situations also occur in the other three ICF Activities and Participation domains. For example, the ICF Activities and Participation domain ‘learning and applying knowledge’ includes “executing a chosen solution, such as resolving a dispute between two people” (d175 Solving problems) [16]. For this item, communication is needed. We therefore included all nine ICF domains. Although domain 3) communication by itself does not describe communicative participation [2, 15], we wanted to ensure our results can directly build on Eadie et al. [2]. Therefore, we did include this domain in line with Eadie et al. and any items that fall within this domain (such as “asking questions in a conversation”). Each of the ICF Activities and Participation third level items (three-digit codes, such as “d720 Relating with strangers” of the ICF interpersonal interactions and relationships domain) and their description (“Engaging in temporary contacts and links with strangers for specific purposes, when asking for directions or other information, or making a purchase” [16]) were used in the labelling process. Two authors (NW and LE) independently classified the items into the different domains using Microsoft Excel [36]. When in doubt, a third author was consulted (CT: research expert in PROM development). The authors discussed their results until consensus was reached.

An overview was made of the classification of items in ICF Activities and Participation domains per PROM which provides insight into the extent to which these items may be suitable for extending the CPIB.

## Results

### Screening abstracts, articles, and outcome measure databases

**Fig. 1:**
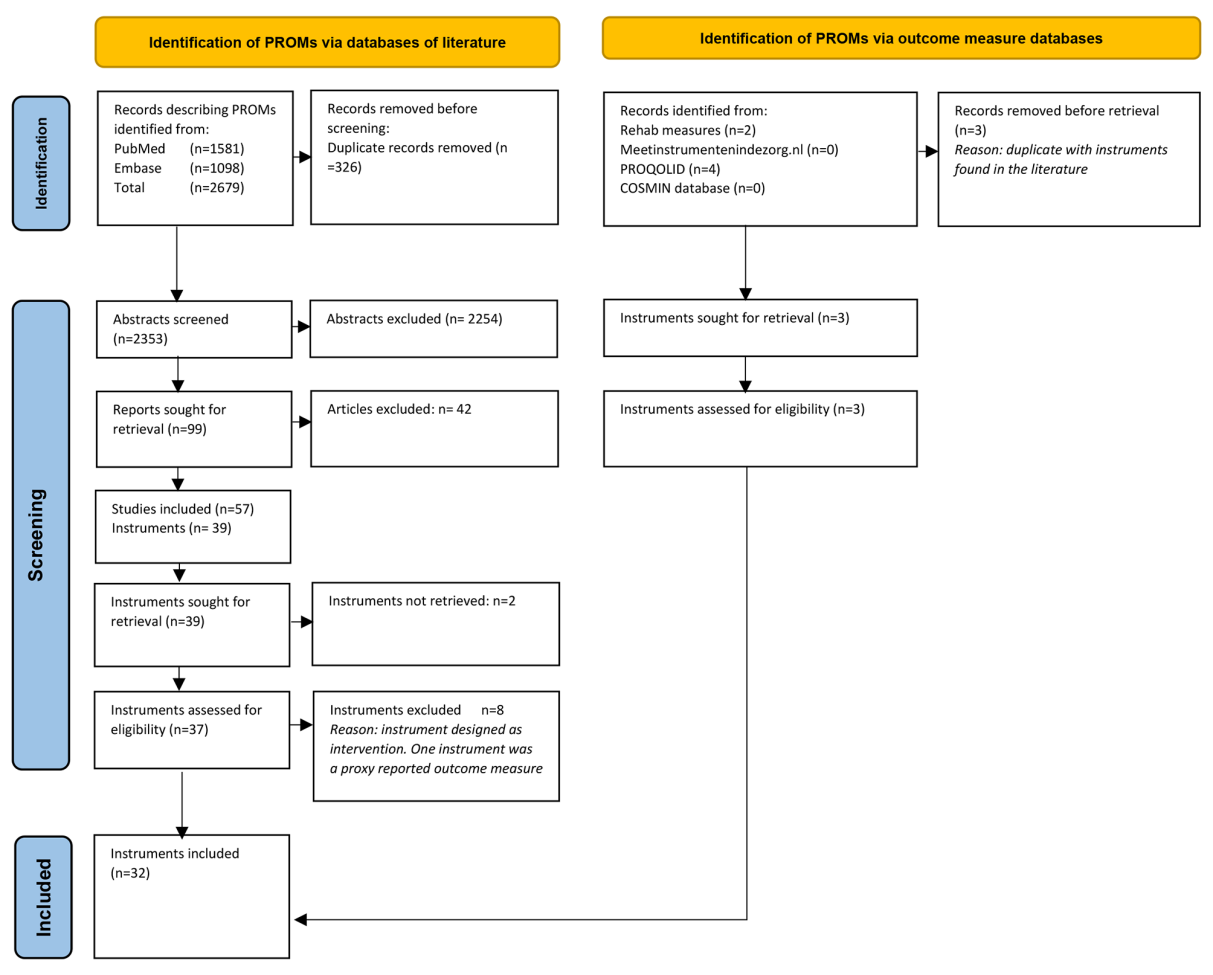
Inclusion of abstracts, articles, and instruments

Two authors (NW and AV) screened 2353 unique abstracts and 99 full-text articles (with an inter-rater reliability of 96.1%). After screening the abstracts and articles, 38 new PROMs (in addition to the CPIB) were included that measure (aspects of) communication according to the authors of the included articles. Ten out of the 38 PROMs were excluded based on further review and three PROMs from the other databases were identified, leading to a final set of 31 new PROMs included. The screening process of abstracts, full-text articles and PROMs is described in Fig. 1.

### Labeling of communicative participation items

The 31 new PROMs contained a total of 909 items. Of these items, 391 (43.0%) were labelled as measuring communicative participation. In Table 2, an example is shown of which items were considered as measuring communicative participation using the Aphasia Communication Outcome Measure (ACOM; [37]). Items 1 and 2 were considered to measure communicative participation while items 3 and 4 did not, since they lack social context. Table 3 describes all 32 identified PROMs, their abbreviations, reference, and number of items measuring communicative participation.

**Table 2:** Example labeling communicative participation items of the Aphasia Communication Outcome Measure (ACOM; [37])

Item	ICF-domain	Measuring communicative participation	Reason for (not) measuring communicative participation
1. ask for information from store employees	6^1^	Yes	A message is send to a natural person, who has the opportunity to respond immediately to the initial communication partner
2. explain your health concerns to your doctor	5^2^	Yes	
3. write a personal letter	7^3^	Yes	
4. understand medicine labels		No	No communicative exchange with a communication partner
5. fill out complex forms		No	A message is send, but not to a natural person, but to an organization or similar.

^*1*^ *ICF-domain: community, social and civic life*

^*2*^ *ICF-domain: self-care*

^*3*^ *ICF-domain: interpersonal interactions and relationships*

**Table 3:** Names of all PROMs included, their abbreviations, reference and percentage of items measuring communicative participation

Name PROM (abbreviation)	Reference	Total Items	Items measuring Communicative Participation (%)	ICF-domains covered (n)								
				1^1^	2^2^	3^3^	4^4^	5^5^	6^6^	7^7^	8^8^	9^9^
Aphasia communication outcome measure (ACOM)	[37]	59	33 (55.9)			17		2	1	12		1
Aphasia Impact Questionnaire (AIQ)	[38]	21	5 (23.8)							5		
Assessment of Language Use in Social Contexts for Adults (ALUSCA)	[39]	91	91 (100)			61				22	4	4
Communication and language assessment questionnaire for persons with multiple sclerosis (CLAMS)	[40]	11	0 (0)									
Communication confidence rating scale for Aphasia (CCRSA)	[41]	10	3 (30)			2					1	
Communication Disability Profile (CDP)	[42]	35	13 (37.1)			5				8		
Communication Outcome after Stroke (COAST)	[43]	20	7 (35)			5				2		
Communicative Activities Checklist (COMACT)	[44]	45	14 (31.1)			6			1	6		1
Communicative Participation Item Bank (CPIB)	[20]	46	46 (100)			19		1	2	21		3
Conversation and Communication Questionnaire for People with Aphasia (CCQA)	[45]	14	4 (28.6)			4						
Dysarthria Impact Profile (DIP)	[46]	52	15 (28.8)			9			1	5		
Emotional Communication in Hearing Questionnaire (EMO-CHeQ)	[47]	16	7 (43.8)			6				1		
Experienced Communication in Dementia Questionnaire (ECD-P)	[48]	24	15 (62.5)			8				7		
Freiburg Questionnaire of linguistic pragmatics (FQLP)	[49]	11	2 (18.2)			1					1	
HDQLIFE Speech Difficulties	[50]	27	6 (22.2)			6						
Hearing Screening of the Elderly (SHSE)	[51]	20	10 (50)			8				1		1
Speech handicap index (SHI)	[52]	30	9 (30)			8				1		
Living with Dysarthria (LwD)	[53]	50	19 (38)			12			1	6		
Neuro-QoL Scale v1.0 - Communication	[54]	5	2 (40)							2		
Overall Assessment of the Speaker’s Experience of Stuttering - Adults (OASES-A)	[55]	100	32 (32)			15				7	6	4
Quality of Life in the Speaker with Dysarthria (QOL-DyS)	[56]	40	25 (62.5)			16				8	1	
Quality of life questionnaire Aphasia (QLQA)	[57]	37	14 (37.8)			12				2		
Satisfaction with Communication in Everyday Speaking Situations Scale (SCESS)	[58]	1	1 (100)			1						
Self-efficacy for situational communication management questionnaire (SESMQ)	[59]	20	16 (80)					1	1	12	1	1
Stroke Communication Scale (SCS)	[60]	35	5 (14.3)			4				1		
Stuttering Generalization Self Measure (SGSM)	[61]	18	9 (50)			1				7	1	
Tinnitus and Hearing Survey (THS)	[62]	10	3 (30)			3						
Tinnitus Functional Index (TFI)	[63]	25	2 (8)			2						
Tinnitus Primary function Questionnaire (TPF)	[64]	20	0 (0)									
Traumatic Brain Injury - Quality of life Communication Item Bank (TBI-QOL communication)	[65]	31	17 (54.8)			11				6		
Verbal Activity Log (VAL)	[66]	12	11 (91.7)			6				3		2
Vocal Fatigue Index (VFI)	[67]	19	1 (5.3)							1		

^1^ Learning and applying knowledge

^2^ General tasks and demands

^3^ Communication

^4^ Mobility

^5^ Self-care

^6^ Domestic life

^7^ Interpersonal interactions and relationships

^8^ Major life areas

^9^ Community, social and civic life

### Classification of items in subdomains

**Fig. 2:**
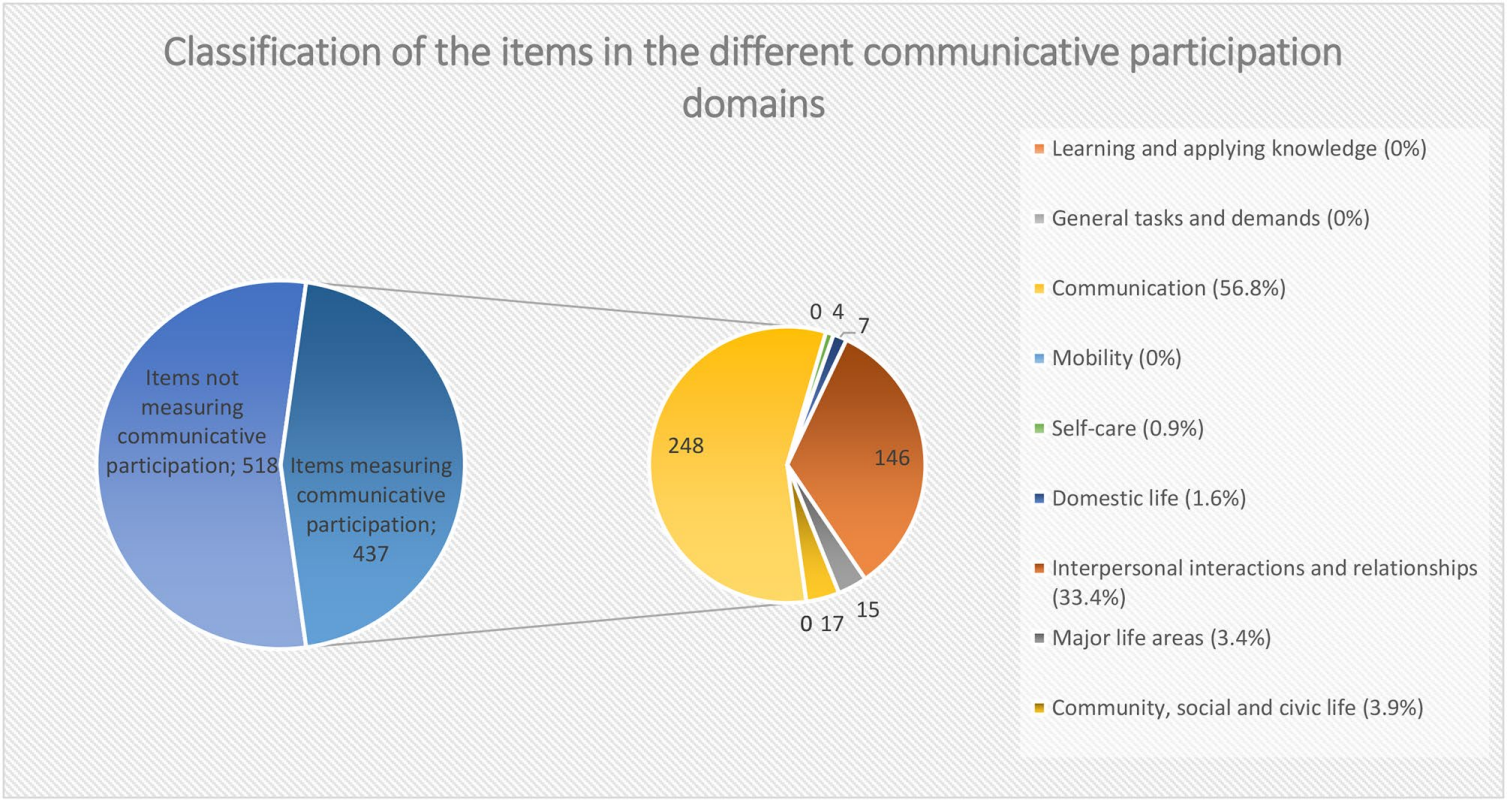
classification of the items in the different communicative participation domains

The 391 communicative participation-items of the 31 new PROMs plus the 46 items from the CPIB (437 items in total) were classified into the different communicative participation domains corresponding to the ICF Activities and Participation domains. The majority of items were classified in the communication domain (n = 248, 56.8%) followed by the interpersonal interactions and relationships domain (n = 146, 33.4%). 3.9% (n = 17) of the items were classified in the community, social and civic life domain and 3.4% (n = 15) in the domain of major life areas. Based on the wording of items, none of the items are related to the domain ‘learning and applying knowledge’, ‘general tasks and demands’ and ‘mobility’. Figure 2 shows the classification in communicative participation domains for all items. For the classification in domains per PROM, see Tables 3 and Appendix 2. For the items included for each domain, see Appendix 3.

## Discussion

This study describes a review of PROMs developed since 2006 to identify items suitable for inclusion in the CPIB. We identified 31 new PROMs targeting communication in adults with different communication problems, that were developed after 2006, and identified 391 items measuring communicative participation.

We argued that a PROM aimed at measuring communicative participation in all its breadth, should include questions covering all ICF Activity and Participation domains [2]. All items of the CPIB were labelled as measuring communicative participation in this study, but the instrument does not cover the domains (1) learning and applying knowledge, (2) general tasks and demands, 4) mobility and 8) major life areas. In addition, it is unclear whether the domains self-care, domestic life and community, social and civic life are covered comprehensively in the CPIB, as they are covered by only one, two and three questions, respectively. It could be that items relevant to those domains were removed in the IRT analysis because of, for example, similar item difficulty, poor item fit, or local dependence [30]. We identified an impressive 391 new items that capture (aspects of) communicative participation and can possibly be added to the CPIB (after excluding overlapping items and if they were to fit the underlying IRT model) to ensure that this instrument fully captures the construct of communicative participation. However, of the items that we identified as covering communicative participation, none are related to the domain ‘learning and applying knowledge’, ‘general tasks and demands’ and ‘mobility’. Examples of items that could be added to cover these domains are: ‘resolving a dispute between two people’ (d175 Solving Problems), ‘communicating while undertaking multiple tasks in a group’ (d220 Undertaking multiple tasks) and ‘communicating with public transportation staff’ (d470 using transportation) respectively [16]. Also, as stated before, the context of communicative participation is changing rapidly, and new communicative participation problems are likely to evolve with change in (digital) communication means. In addition, the construct communicative participation is a comprehensive and multifaceted construct. On the one hand, it consists of the different facets of communication: it may take place verbally through spoken and written language, as well as non-verbally. On the other hand, it consists of the facets of participation, where the different life situations may take place for a defined social goal (e.g., establishing relationships), a function/role (e.g., job-related), and/or in a particular context (e.g., in a restaurant) [2]. To ensure an item bank contains all items relevant for the construct of interest, it is important to involve the target population for which the items are intended [68, 69]. Therefore, we also initiated a concept elicitation study in people with speech, language, hearing, and voice difficulties, to possibly identify new content for extending and updating the CPIB [70].

As described in the method section, we included domain 3) communication in the labelling process of items into different ICF Activity and Participation domains. Eadie et al. [2] concluded that these items need to be revised if they are to be included in a Communicative Participation item bank, by adding different social goals, functions, or life situations to reflect the ‘participation’ component of communicative participation. Our research however shows that the CPIB does include general communication items (e.g., “asking questions in a conversation” or “communicating in a small group of people”). General communication of course is a prerequisite for communicative participation, and communication always occurs in life situations. We question whether these general communication items formulated without the explicit participation contexts, are understood in a consistent manner by people with different communicative problems, and whether they are distinctive enough on an Item Response Theory calibrated scale. It may be worth reconsidering the inclusion of these more activity related items in the CPIB.

Some limitations of our study should be acknowledged. We included only PROMs with at least some available information on the measurement properties, as this increased the likelihood of including relevant and reliable items. However, items from PROMs not validated at all, could also have been relevant. In addition, as our review focused on the individual items of existing PROMS, we did not assess the quality of the studies included, or the quality of the PROMs as a whole. Furthermore, only English and Dutch articles and PROMs were included. Therefore, other relevant PROMs with communicative participation items developed in other languages may not have been included in this study.

## Conclusion

We identified 31 new PROMs that include a total of 391 items measuring communicative participation that could be used to extend the CPIB. We found items in domains that are already present in the CPIB, but also items that relate to new domains, which could be considered for inclusion in the item bank, to further improve the comprehensiveness and applicability of the CPIB.

## Electronic supplementary material

Below is the link to the electronic supplementary material.

Supplementary Material 1

Supplementary Material 2

Supplementary Material 3

## Data Availability

The datasets used and/or analysed during the current study are available from the corresponding author on reasonable request.

## List of abbreviations

ACOM: Aphasia communication outcome measure
CPIB: Communicative Participation Item Bank
ICF: International Classification of Functioning, Disability and Health
PROM: Patient Reported Outcome Measure
SCESS: Satisfaction with Communication in Everyday Speaking Situations Scale
SLT: Speech and Language Therapist
IRT: Item Response Theory

## Footnotes

1 The first search was performed from 2006 up to June 2020. An extra search was performed from 2020 up to December 2020. For this search, a similar search string was developed for Pubmed, since Pubmed received an update. For search #3 (see Appendix 1) we used [Major] instead of [Mesh] to limit the number of results.

2 This search strategy is developed for Pubmed. Search strategies and search strings for other databases are similar and available upon request from the first author of this article.
