# Supplementary material for: Further development in measuring communicative participation: identifying items to extend the applicability of the communicative participation item bank
Source: J Patient Rep Outcomes. 2023 May 26;7:49. doi: 10.1186/s41687-023-00586-8 (PMC10219900; doi:10.1186/s41687-023-00586-8)
Supplement: Supplementary file 2 — Supplementary Material 2 [file 41687_2023_586_MOESM2_ESM.docx]

| Appendix 2: Names of all PROMs included, their abbreviations, reference and percentage of items measuring communicative participation | | | | | | | | | | | | |
| --- | --- | --- | --- | --- | --- | --- | --- | --- | --- | --- | --- | --- |
| Name PROM (abbreviation) | Reference | Total Items | Items measuring Communicative Participation (%) | ICF-domains covered (n) | | | | | | | |  |
|  |  |  |  | 1^1^ | 2^2^ | 3^3^ | 4^4^ | 5^5^ | 6^6^ | 7^7^ | 8^8^ | 9^9^ |
| Aphasia communication outcome measure (ACOM) | [37] | 59 | 33 (55.9) |  |  | 17 |  | 2 | 1 | 12 |  | 1 |
| Aphasia Impact Questionnaire (AIQ) | [38] | 21 | 5 (23.8) |  |  |  |  |  |  | 5 |  |  |
| Assessment of Language Use in Social Contexts for Adults (ALUSCA) | [39] | 91 | 91 (100) |  |  | 61 |  |  |  | 22 | 4 | 4 |
| Communication and language assessment questionnaire for persons with multiple sclerosis (CLAMS) | [40] | 11 | 0 (0) |  |  |  |  |  |  |  |  |  |
| Communication confidence rating scale for Aphasia (CCRSA) | [41] | 10 | 3 (30) |  |  | 2 |  |  |  |  | 1 |  |
| Communication Disability Profile (CDP) | [42] | 35 | 13 (37.1) |  |  | 5 |  |  |  | 8 |  |  |
| Communication Outcome after Stroke (COAST) | [43] | 20 | 7 (35) |  |  | 5 |  |  |  | 2 |  |  |
| Communicative Activities Checklist (COMACT) | [44] | 45 | 14 (31.1) |  |  | 6 |  |  | 1 | 6 |  | 1 |
| Communicative Participation Item Bank (CPIB) | [20] | 46 | 46 (100) |  |  | 19 |  | 1 | 2 | 21 |  | 3 |
| Conversation and Communication Questionnaire for People with Aphasia (CCQA) | [45] | 14 | 4 (28.6) |  |  | 4 |  |  |  |  |  |  |
| Dysarthria Impact Profile (DIP) | [46] | 52 | 15 (28.8) |  |  | 9 |  |  | 1 | 5 |  |  |
| Emotional Communication in Hearing Questionnaire (EMO-CHeQ) | [47] | 16 | 7 (43.8) |  |  | 6 |  |  |  | 1 |  |  |
| Experienced Communication in Dementia Questionnaire (ECD-P) | [48] | 24 | 15 (62.5) |  |  | 8 |  |  |  | 7 |  |  |
| Freiburg Questionnaire of linguistic pragmatics (FQLP) | [49] | 11 | 2 (18.2) |  |  | 1 |  |  |  |  | 1 |  |
| HDQLIFE Speech Difficulties | [50] | 27 | 6 (22.2) |  |  | 6 |  |  |  |  |  |  |
| Hearing Screening of the Elderly (SHSE) | [51] | 20 | 10 (50) |  |  | 8 |  |  |  | 1 |  | 1 |
| Speech handicap index (SHI) | [52] | 30 | 9 (30) |  |  | 8 |  |  |  | 1 |  |  |
| Living with Dysarthria (LwD) | [53] | 50 | 19 (38) |  |  | 12 |  |  | 1 | 6 |  |  |
| Neuro-QoL Scale v1.0 - Communication | [54] | 5 | 2 (40) |  |  |  |  |  |  | 2 |  |  |
| Overall Assessment of the Speaker's Experience of Stuttering - Adults (OASES-A) | [55] | 100 | 32 (32) |  |  | 15 |  |  |  | 7 | 6 | 4 |
| Quality of Life in the Speaker with Dysarthria (QOL-DyS) | [56] | 40 | 25 (62.5) |  |  | 16 |  |  |  | 8 | 1 |  |
| Quality of life questionnaire Aphasia (QLQA) | [57] | 37 | 14 (37.8) |  |  | 12 |  |  |  | 2 |  |  |
| Satisfaction with Communication in Everyday Speaking Situations Scale (SCESS) | [58] | 1 | 1 (100) |  |  | 1 |  |  |  |  |  |  |
| Self-efficacy for situational communication management questionnaire (SESMQ) | [59] | 20 | 16 (80) |  |  |  |  | 1 | 1 | 12 | 1 | 1 |
| Stroke Communication Scale (SCS) | [60] | 35 | 5 (14.3) |  |  | 4 |  |  |  | 1 |  |  |
| Stuttering Generalization Self Measure (SGSM) | [61] | 18 | 9 (50) |  |  | 1 |  |  |  | 7 | 1 |  |
| Tinnitus and Hearing Survey (THS) | [62] | 10 | 3 (30) |  |  | 3 |  |  |  |  |  |  |
| Tinnitus Functional Index (TFI) | [63] | 25 | 2 (8) |  |  | 2 |  |  |  |  |  |  |
| Tinnitus Primary function Questionnaire (TPF) | [64] | 20 | 0 (0) |  |  |  |  |  |  |  |  |  |
| Traumatic Brain Injury - Quality of life Communication Item Bank (TBI-QOL communication) | [65] | 31 | 17 (54.8) |  |  | 11 |  |  |  | 6 |  |  |
| Verbal Activity Log (VAL) | [66] | 12 | 11 (91.7) |  |  | 6 |  |  |  | 3 |  | 2 |
| Vocal Fatigue Index (VFI) | [67] | 19 | 1 (5.3) |  |  |  |  |  |  | 1 |  |  |

^1^ Learning and applying knowledge

^2^ General tasks and demands

^3^ Communication

^4^ Mobility

^5^ Self-care

^6^ Domestic life

^7^ Interpersonal interactions and relationships

^8^ Major life areas

^9^ Community, social and civic life
